# Prognosis of comatose patients with reduced EEG montage by combining quantitative EEG features in various domains

**DOI:** 10.3389/fnins.2023.1302318

**Published:** 2023-12-08

**Authors:** Tao Tao, Shiqi Lu, Nan Hu, Dongyang Xu, Chenyang Xu, Fajun Li, Qin Wang, Yuan Peng

**Affiliations:** ^1^Intensive Care Unit, The First People’s Hospital of Kunshan, Kunshan Affiliated Hospital of Jiangsu University, Kunshan, Jiangsu, China; ^2^Emergency Department, The First Affiliated Hospital of Soochow University, Suzhou, Jiangsu, China; ^3^School of Electronics and Information Engineering, Soochow University, Suzhou, Jiangsu, China; ^4^Center for Intelligent Acoustics and Signal Processing, Huzhou Institute of Zhejiang University, Huzhou, China

**Keywords:** coma prognosis, electroencephalography, frontoparietal network, limited frontoparietal EEG montage, functional connectivity

## Abstract

**Objective:**

As the frontoparietal network underlies recovery from coma, a limited frontoparietal montage was used, and the prognostic values of EEG features for comatose patients were assessed.

**Methods:**

Collected with a limited frontoparietal EEG montage, continuous EEG recordings of 81 comatose patients in ICU were used retrospectively. By the 60-day Glasgow outcome scale (GOS), the patients were dichotomized into favorable and unfavorable outcome groups. Temporal-, frequency-, and spatial-domain features were automatically extracted for comparison. Partial correlation analysis was applied to eliminate redundant factors, and multiple correspondence analysis was used to explore discrimination between groups. Prognostic characteristics were calculated to assess the performance of EEG feature-based predictors established by logistic regression. Analyses were performed on all-patients group, strokes subgroup, and traumatic brain injury (TBI) subgroup.

**Results:**

By analysis of all patients, raised burst suppression ratio (BSR), suppressed root mean square (RMS), raised power ratio of β to α rhythm (β/α), and suppressed phase-lag index between F3 and P4 (PLI [F3, P4]) were associated with unfavorable outcome, and yielded AUC of 0.790, 0.811, 0.722, and 0.844, respectively. For the strokes subgroup, the significant variables were BSR, RMS, θ/total, θ/δ, and PLI (F3, P4), while for the TBI subgroup, only PLI (F3, P4) was significant. BSR combined with PLI (F3, P4) gave the best predictor by cross-validation analysis in the all-patients group (AUC = 0.889, 95% CI: 0.819–0.960).

**Conclusion:**

Features extracted from limited frontoparietal montage EEG served as valuable coma prognostic tools, where PLI (F3, P4) was always significant. Combining PLI (F3, P4) with features in other domains may achieve better performance.

**Significance:**

A limited-montage EEG coupled with an automated algorithm is valuable for coma prognosis.

## Highlights

- A limited frontoparietal EEG montage was employed for EEG monitoring.
- A combination of EEG features was used to build predictors for coma outcome in ICU.
- Significant variables were found in temporal, frequency, and spatial domains.
- Burst suppression ratio combined with PLI between F3 and P4 formed the best predictor.
- EEG monitoring for frontoparietal network can contribute to coma prognosis.

## 1 Introduction

Patients with acquired brain injuries (ABIs) often suffer from severe disorders of consciousness (DoC), whose preceding state is coma, an acute state lasting up to 2–4 weeks or even longer (Owen, 2015). The Glasgow Coma Scale (GCS) is a conventional assessment tool for comatose patients, including three sub-scales: eye-opening (E), verbal response (V), and motor response (M). A patient is considered in coma only when E = 1, V ≤ 2, and M ≤ 4 are satisfied simultaneously (Zubler et al., 2015), and in the meantime, the overall GCS = E + V + M ≤ 7 is given. Otherwise, the patient is no longer in a coma state but has turned to a post-coma DoC state, including unresponsive wakefulness syndrome (UWS), minimally conscious state (MCS), or EMCS (emerged from minimally conscious state), as assessed by the Coma Recovery Scale–Revised (CRS-R; Giacino et al., 2004) instead. Early prognosis of comatose patients in intensive care unit (ICU) has the potential to assist clinical decision-making for further treatment. Though the frequently used GCS is based on standardized objective examination findings, it needs expert interpretation, and it does not consider the underlying pathophysiology, the trajectory of neuro-recovery, or the presence of various confounding factors. Hence, developing a new coma outcome predictor based on continuous neurophysiological monitoring coupled with an automated algorithm has drawn much attention in recent years.

Electroencephalography (EEG) is known for its advantage in monitoring the overall functionality of the cerebral cortex and the neural response to external stimuli. When applied in neuro-prognostication for comatose patients, continuous bedside EEG monitoring offers the possibility of objective assessments of the trajectory of neuro-recovery with high temporal resolution (Young, 2000). EEG signals include evoked potentials and resting-state EEG. Evoked potentials used in coma prognosis include brainstem auditory evoked potential (BAEP; Rothstein, 2000), mid-latency auditory evoked potential (MLAEP; Tsurukiri et al., 2013), somatosensory evoked potential (SEP; Zhang et al., 2015), and auditory steady-state response (ASSR; Chen et al., 2020). Compared with evoked potentials depending on additional stimulation and synchronization devices, resting-state EEG corresponds to recording the patients’ spontaneous EEG only, which is more suitable for long-term monitoring.

The American Clinical Neurophysiology Society (ACNS) recommended some standardized EEG descriptors (terminologies) to assess the patients in ICU (Hirsch et al., 2021), including continuity, voltage, frequency, symmetry, organization of an anterior/posterior gradient of the background activity, presence of reactivity, spontaneous variability of the background activity, and occurrence of epileptic discharges. Most of the ACNS EEG descriptors can be obtained from resting-state EEG recordings reviewed visually by experts or processed automatically by algorithms. In Hofmeijer et al. (2016), EEG of consecutive comatose patients after cardiac arrest was reviewed to be classified as isoelectric, low-voltage, epileptiform, burst suppression, diffusely slowed, or normal, and the classified states were applied in coma outcome prediction. ACNS EEG descriptors were used in Benarous et al. (2019) to predict outcome of postanoxic coma and showed the necessity of standardized methods of evaluating EEG parameters. In Scarpino et al. (2020), ACNS EEG descriptors combined with SEPs recorded at 12 and 72 h from resuscitation were used for predicting 6-month neurological outcome in comatose patients after cardiac arrest. Background activity, the presence of rhythmic or periodic patterns, and the reactivity of ACNS EEG descriptors were used in Guedes et al. (2020) to illustrate that they can be reliable predictors for poor neurological outcome as well as death. ACNS EEG descriptors in a reduced EEG montage study in Backman et al. (2020) also showed significant value in assessing comatose cardiac arrest patients. As a weighted sum of resting-state EEG parameters at temporal and frequency domains, ranging from 0 to 100, the bispectral index (BIS) has also been used in prediction of coma outcome (Schnakers et al., 2008).

Most of the EEG features used for coma prognosis, including ACNS EEG descriptors, were derived from temporal domain or frequency domain (Hoedemaekers et al., 2023), and the current trend is to involve deep neural networks with larger datasets (Zheng et al., 2022). In recent years, spatial EEG features have also been explored in this field. Coupling between EEG signals on the left–right axis and on the anterior–posterior axis was measured with four synchronization measures in Zubler et al. (2015) and used in coma prognosis. Based on the similarity of instantaneous frequencies in EEG epochs, link rates (LRs) and link durations (LDs) in the α, δ, and θ bands were calculated for outcome prediction of comatose patients after cardiac arrest (Keijzer et al., 2021). Three functional connectivity metrics, coherence (COH), phase locking value (PLV), and mutual information (MI), were calculated in 19-channel EEGs at 12, 24, and 48 h after cardiac arrest (Carrasco-Gómez et al., 2021), and machine learning techniques were used to combine them in a model to predict outcome of postanoxic coma. An intrinsic network reactivity index (INRI; Khanmohammadi et al., 2018) based on whole-brain multi-channel resting-state EEG was formed to study its correlation with the consciousness level of coma patients. Despite the above preliminary studies, the combination of the temporal-, frequency-, and spatial-domain features of resting-state EEG has seldom been used in prognostic research for comatose patients.

Relative to the neurologic examination-based neuro-prognostication scales, EEG is time-intensive, much more expensive, and not available in much of the resource-limited world. Therefore, conventional full-montage-based EEG monitoring combined with expert visual interpretation or supervised quantification techniques needs to be improved for coma prognosis. In this context, it is meaningful to assess the prognostic value of a limited-montage EEG coupled with an automated algorithm. In Backman et al. (2020), with a reduced EEG montage including six channels (F3, T3, P3, F4, T4, and P4), visually interpreted ACNS EEG descriptors yielded high prognostic performance for postcardiac arrest comatose patients. In recent years, the anterior forebrain mesocircuit (Fridman et al., 2014) and the frontoparietal network (Wu et al., 2015) have been consistently implicated in circuit mechanisms underlying recovery from coma (Edlow et al., 2021). Inspired by these circuit mechanisms underlying the restoration of cerebral activity during recovery from coma, we only reserve frontal and parietal electrodes in the reduced EEG montage used in Backman et al. (2020). The quantitative EEG features in temporal domain, frequency domain, and spatial domain can be extracted automatically from this limited-montage EEG. The prognostic value of a combination of quantitative EEG features in various domains would then be assessed in predicting outcomes of comatose patients with ABIs in ICU. At last, ancillary neuro-prognostication methods would be developed for all comatose population or patients in specific subgroups.

## 2 Materials and methods

### 2.1 Study design

This study was a retrospective one. Comatose patients with ABIs admitted to the ICU of the First People’s Hospital of Kunshan from 29 October 2019 to 7 April 2021, who underwent multi-channel EEG monitoring, were considered. Inclusion criteria were: (1) The patients were in coma, i.e., GCS ≤ 7 with E = 1, V ≤ 2, and M ≤ 4; (2) EEG monitoring was conducted within 4 weeks after admission, including at least four frontoparietal channels: F3, F4, P3, P4, and the EEG recording lasted for at least 30 min; (3) EEG monitoring was initiated at or later than 24 h after sedating medications (André-Obadia et al., 2018); (4) The 60-day Glasgow outcome scale (GOS; Jennett and Bond, 1975) of the patient could be obtained by follow-up; (5) The patient was not diagnosed as brain dead before or during EEG monitoring, which would also show isoelectric (<2 μV) EEG waveform; (6) The patient did not regain consciousness during EEG monitoring. Qualified patients of both sexes were eligible for the study. According to the above criteria, a total of 81 comatose patients were included in the study cohort, and their 60-day GOS scores were obtained in follow-up. Ranked from 1 to 5, the GOS provides a measurement of post-coma outcome: 1 = death; 2 = vegetative state or severe disability; 3 = moderate disability, able to follow commands but unable to live independently; 4 = able to live independently but unable to return to work or school; 5 = fully recovered. Among the 81 patients who participated, we had GOS = 1 (*n* = 39), GOS = 2 (*n* = 20), GOS = 3 (*n* = 10), GOS = 4 (*n* = 7), and GOS = 5 (*n* = 5). According to the GOS, the patients were dichotomized into favorable (GOS ≥ 3) and unfavorable (GOS ≤ 2) outcome groups. Finally, there were 22 patients in the favorable outcome group and 59 patients in the unfavorable outcome group.

In this study, the determination of conducting quantitative EEG monitoring was given by each patient’s physician, instead of our study purpose. Our study did not influence the treatment or decision to withdraw life-sustaining therapy. Since EEG monitoring is part of standard care in our ICU, the need for informed consent for EEG measurements and follow-up by telephone interview was waived. The study protocol was reviewed and approved by the Medical Ethics Committee of the First People’s Hospital of Kunshan (Approval No. 00012098), which ensured that our study was conducted in accordance with the ethical guidelines of the Declaration of Helsinki.

### 2.2 EEG recording and feature extraction

Quantitative EEG monitoring was conducted using a multi-lead EEG device (Cadwell Industries, Kennewick, WA, United States). A limited frontoparietal EEG montage was used, where F3, F4, P3, and P4 were used as EEG recording electrodes, and Cz was reference. The sampling rate was 250 Hz, and high-quality EEG recording lasted for at least 30 min. Considering the particularity of sedation and analgesia in the treatment of critically ill patients, and especially analgesic drugs being the basis of medication, we used a combination regimen of short-acting low-dose remifentanil and low-dose dexmedetomidine for all patients. It was known that sedation may lead to alteration of some neurophysiological markers (André-Obadia et al., 2018; Benghanem et al., 2022), including decreased voltage, decreased slow wave, and raised fast rhythms. In our ICU, all EEG monitoring were initiated at or later than 24 h after short-acting low-dose sedating medications and ended before next sedating medications. The occurrence of epileptic discharges, which was mainly focused on non-convulsive status epilepticus, was also visually interpreted and recorded by physicians. The physicians were not blinded to the EEG as they determined the EEG monitoring and had to treat epileptic seizures.

**Figure 1:**
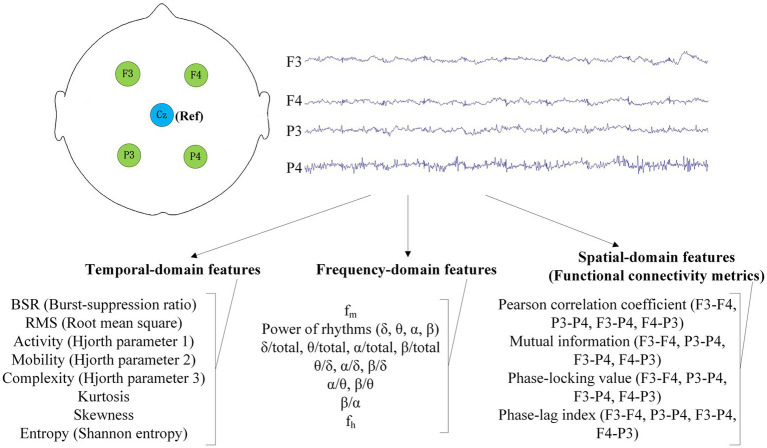
EEG recording and feature extraction in this study.

Each patient’s EEG recording was partitioned into 2-min non-overlapping epochs. EEG in each epoch underwent baseline calibration, bandpass filtering, and trajectory rejection. A 0.1–40 Hz forward–backward four-order Butterworth filter was used to eliminate noise and interference. For rejection of trajectories such as abnormal movements, an epoch would be discarded if there existed a point whose density was >200 μV. For each epoch, 8 temporal features, 16 frequency features, and 24 spatial features were calculated. Temporal features or frequency features were calculated for all channels, and the median value of each kind of feature across all channels was used. The features among all reserved epochs were averaged to give the final variables for coma prognosis study. The diagram of EEG recording and feature extraction is displayed in Figure 1. EEG preprocessing and feature extraction were performed using MATLAB 2022a and the EEGLAB toolbox.

The extracted temporal features were: BSR (burst suppression ratio), RMS (root mean square), activity, mobility, complexity, kurtosis, skewness, and entropy. BSR was defined as the ratio of duration of EEG in suppression state (≤5 μV) to the total duration in one epoch (Nagaraj et al., 2018). If the EEG samples in one epoch are denoted as $x_{n},\;n=1,\;2,\;\ldots ,\;N$ , where N is the number of samples in one epoch, RMS = $\sqrt{\mathrm{var}\left(x_{n}\right)}=\sqrt{\overset{N}{\underset{n=1}{\sum }}{\left(x_{n}-\bar{x}\right)}^{2}/N}$ . Activity, mobility, and complexity are Hjorth parameters (Hjorth, 1970), given by activity = $\mathrm{var}\left(x_{n}\right)$ , mobility = $\sqrt{\mathrm{var}\left({x_{n}}^{\prime }\right)/\mathrm{var}\left(x_{n}\right)}$ , and complexity = $\mathrm{mobility}\left({x_{n}}^{\prime }\right)/\mathrm{mobility}\left(x_{n}\right)$ , respectively. Kurtosis and skewness are frequently used higher-order statistics. Entropies were usually known as chaos features, but in this study, they were still categorized as temporal features, and Shannon entropy was used. The extracted frequency features were: the frequency corresponding to the maximum frequency component (f_m_), power of δ rhythm (0.5–4 Hz), power of θ rhythm (4–8 Hz), power of α rhythm (8–13 Hz), power of β rhythm (13–30 Hz), power ratios (δ/total, θ/total, α/total, β/total, θ/δ, α/δ, β/δ, α/θ, β/θ, β/α), and the upper cutoff frequency (f_h_). The spatial features used functional connectivity metrics, including Pearson correlation coefficient (PCC), mutual information (MI), phase locking value (PLV), and phase-lag index (PLI) for each couple of channels. If *x* and *y* denote two EEG channels, $PCC\left(x,y\right)=\overset{N}{\underset{n=1}{\sum }}\left(x_{n}-\bar{x}\right)\left(y_{n}-\bar{y}\right)/\left(N\sqrt{\mathrm{var}\left(x_{n}\right)\mathrm{var}\left(y_{n}\right)}\right)$ , $MI\left(x,y\right)=H_{x}+H_{y}-H_{xy}$ , $PLV\left(x,y\right)=\left|\langle e^{i\varphi_{x,y}\left(t\right)}\rangle \right|$ , and $PLI\left(x,y\right)=\left|\langle \mathrm{sign}\varphi_{x,y}\left(t\right)\rangle \right|$ , where $H_{x}$ , $H_{y}$ , and $H_{xy}$ denote entropy of *x* channel, entropy of *y* channel, and cross-entropy of *x* channel and *y* channel, respectively, $\varphi_{x,y}\left(t\right)$ denotes difference of unwrapped phases between *x* channel and *y* channel, and $\langle \cdot \rangle$ denotes expectation. In the existing EEG-based coma prognosis studies that exploited functional connectivity metrics (Zubler et al., 2015; Khanmohammadi et al., 2018; Carrasco-Gómez et al., 2021; Keijzer et al., 2021), a common reference or global average reference was used. As a limited montage is used in this study, we chose a common reference Cz for all four frontoparietal channels. It can be proved theoretically that, with a common reference, information of interaction between two coupled channels can still be derived, though attenuated by some interference terms.

### 2.3 Statistical analysis

To compare demographical or extracted feature variables in two patient groups, Fisher’s exact test or χ^2^ test was used for categorical variables (presented as numbers [percentage]), and Student’s *t*-test or Mann–Whitney *U* test was performed for continuous variables, where the significance level was set at *p* = 0.05. The Benjamini–Hochberg correction of false discovery rate was employed for all EEG features. For continuous variables, Shapiro–Wilk test and Levene test were performed first to determine if the variable met normal distribution and homogeneity of variance, respectively. If a variable met normal distribution with homogeneous variance (presented as mean ± SD), the *t*-test was used; otherwise (presented as median [quartile]), the Mann–Whitney *U* test was used. Partial correlation analysis was further applied to eliminate redundant factors. A multiple correspondence analysis (MCA) was used to explore discrimination between variables in two groups. Logistic regression was used to establish prognosis models for a single EEG feature-based predictor or a combination of multiple predictors in various domains. Area under the receiver operating characteristic curve (AUC-ROC) was calculated to assess the performances of the established prognosis models. Prognostic characteristics including sensitivity (Sen), specificity (Spec), positive predictive value (PPV), negative predictive value (NPV), and false positive rate for predicting unfavorable outcome by built predictors were also calculated, where the cutoff probability was set as 0.5. All statistical analyses were conducted using SPSS Statistics 27 (IBM Corp., Armonk, NY, United States).

## 3 Results

Results in comparison of baseline characteristics between groups are displayed in Table 1. The baseline characteristics consist of sex, age, GCS evaluated during EEG recording, etiology, timing of EEG recording after brain injury, and occurrence of epileptic discharges. Among all patients in this study, the etiology included hemorrhagic stroke, ischemic stroke, traumatic brain injury (TBI), postcardiac arrest, and others (intoxication, tumor, pulmonary encephalopathy, etc.). From Table 1, it was found that none of the baseline characteristics showed significance.

**Table 1:** Baseline characteristics of the study population.

Variable	Favorable outcome (GOS ≥ 3)	Unfavorable outcome (GOS ≤ 2)	Statistical value	Value of *p*
	*n* = 22	*n* = 59		
Sex (female)	6 (27.3%)	14 (23.7%)	0.002 (Fisher)	0.776
Age	49.32 ± 17.844	49.20 ± 17.966	0.026 (*t*)	0.980
GCS	3.5 (3)	3 (1)	0.201 (U)	0.841
Etiology	6/0/11/1/4	19/4/22/9/5	5.101 (χ^2^)	0.292
Time of EEG recording after brain injury (days)	6 (15)	3 (5)	1.626 (U)	0.104
Occurrence of epileptic discharges	2 (9.1%)	2 (3.5%)	0.227 (Fisher)	0.297

The order of five etiology items in the table: hemorrhagic stroke, ischemic stroke, traumatic brain injury (TBI), postcardiac arrest, and others.

### 3.1 Analysis of all patients

**Figure 2:**
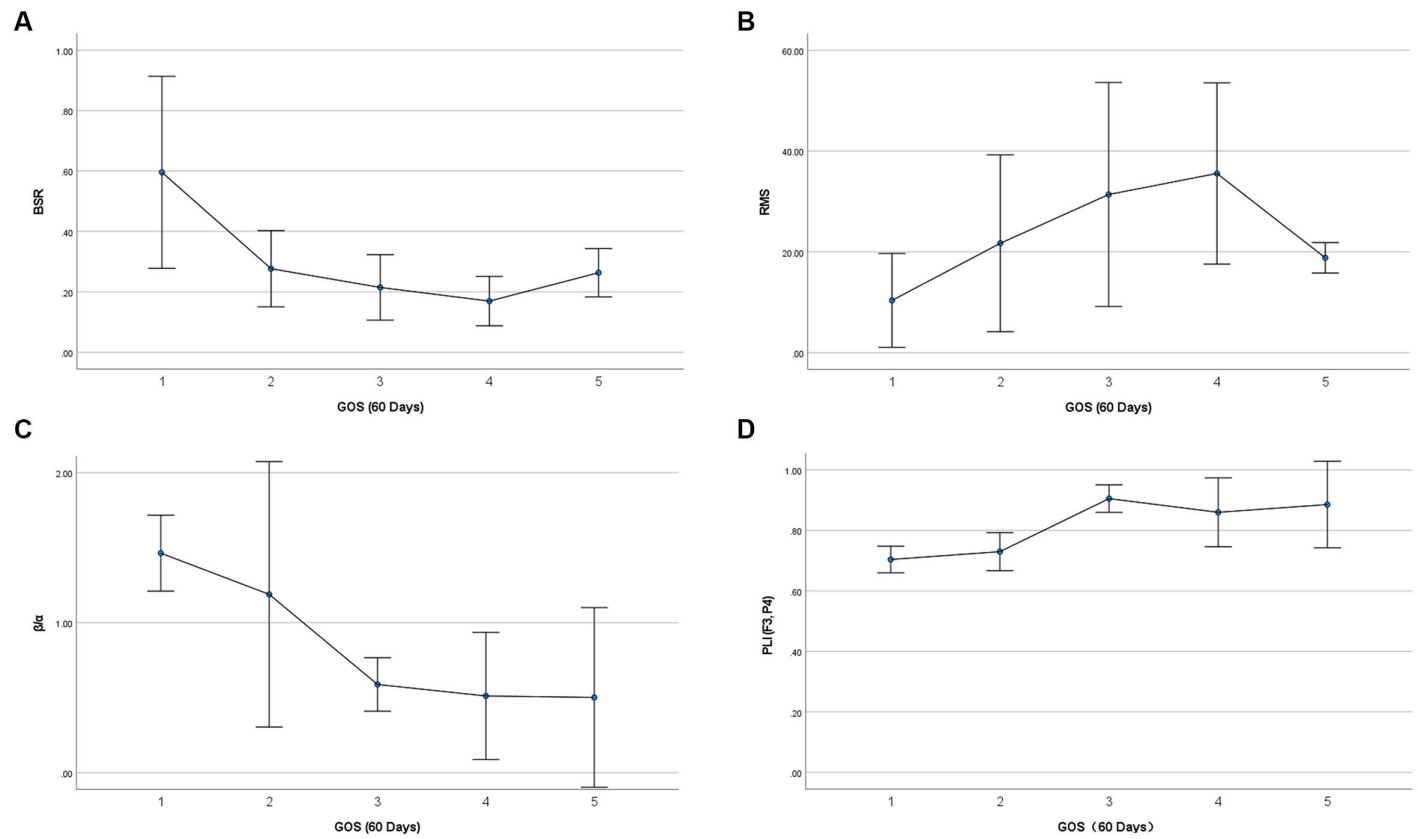
Relationship between selected EEG feature variables and 60-day GOS. Vertical error bars indicate the 95% confidence intervals, and the central points denote mean values.

Comparison of EEG feature variables between the two groups is given in Table 2. It was found that BSR, RMS, activity, powers of δ, θ, α, and β rhythms, β/α, and PLI (F3, P4) showed significant differences between the two groups. RMS, similar to the “Voltage” in ACNS EEG descriptors, showed significance along with other eight EEG features. The EEG voltage amplitude is associated with many EEG features, e.g., rhythm densities extracted by Fourier transform $X\left(f\right)=\int x\left(t\right)e^{-j2\pi ft}dt$ are in direct proportion to RMS. Partial correlation analysis is a useful tool to identify the true relationship between two variables while controlling for the effects of another variable. As the dichotomized outcomes are actually obtained from 60-day GOS, we performed partial correlation analysis between all significant EEG features (except RMS) and 60-day GOS, where RMS was used as controlled variable. Table 3 lists the partial correlation analysis results. It was found that activity and powers of δ, θ, α, and β rhythms were no longer significant, implying that their significance may be due to RMS, and hence these five variables would be pruned in the coma prognosis study. Spearman’s correlations between the remaining significant variables (BSR, RMS, β/α, and PLI [F3, P4]) and 60-day GOS are presented in Table 4. High correlations between all these four variables and 60-day GOS can be found, and the trend of these variables along with the variation of GOS is also displayed in Figure 2 in the form of error bars. Henceforth, in this study, EEG features in temporal domain (BSR and RMS), frequency domain (β/α), and spatial domain (PLI [F3, P4]) would be considered in coma prognosis.

**Table 2:** EEG features in various domains between favorable and unfavorable outcome groups.

Variable	Favorable outcome (GOS ≥ 3)	Unfavorable outcome (GOS ≤ 2)	Statistical value	Value of *p* (after correction)
	*n* = 22	*n* = 59		
BSR	0.187 (0.120)	0.358 (0.563)	−3.992 (U)	**<0.001** ^*^
RMS	22.73 (20.873)	12.58 (15.077)	4.290 (U)	**<0.001** ^*^
Activity	781.67 (1799.21)	145.94 (406.64)	3.961 (U)	**<0.001** ^*^
Mobility	12.69 (7.63)	16.62 (13.54)	−1.688 (U)	0.338
Complexity	4.58 (2.69)	4.24 (2.81)	1.487 (U)	0.445
Kurtosis	3.49 (0.52)	3.45 (1.21)	0.680 (U)	0.738
Skewness	−0.075 (0.282)	−0.016 (0.211)	−1.179 (U)	0.654
Entropy	4.077 (0.193)	4.076 (0.356)	0.074 (U)	0.959
f_m_	0.549 (0.294)	0.479 (0.446)	0.812 (U)	0.699
δ	91.30 (254.51)	24.46 (64.33)	4.141 (U)	**<0.001** ^*^
θ	2.834 (2.817)	0.745 (2.045)	3.589 (U)	**<0.001** ^*^
α	1.283 (1.227)	0.273 (0.624)	3.780 (U)	**<0.001** ^*^
β	0.438 (0.754)	0.140 (0.363)	3.292 (U)	**<0.001** ^*^
δ/total	0.923 (0.075)	0.878 (0.192)	1.593 (U)	0.385
θ/total	0.042 (0.022)	0.048 (0.052)	−0.892 (U)	0.667
α/total	0.016 (0.019)	0.023 (0.050)	−1.210 (U)	0.653
β/total	0.007 (0.014)	0.017 (0.079)	−1.858 (U)	0.328
θ/δ	0.054 (0.045)	0.054 (0.101)	−1.157 (U)	0.642
α/δ	0.018 (0.025)	0.031 (0.080)	−1.295 (U)	0.596
β/δ	0.008 (0.019)	0.022 (0.129)	−1.848 (U)	0.307
α/θ	0.438 (0.313)	0.537 (0.605)	−0.924 (U)	0.740
β/θ	0.197 (0.241)	0.512 (1.234)	−1.752 (U)	0.346
β/α	0.474 (0.582)	0.991 (1.275)	−3.058 (U)	**0.012** ^*^
f_h_	0.887 (0.342)	0.814 (0.474)	0.892 (U)	0.691
PCC (F3, F4)	0.252 ± 0.333	0.335 ± 0.289	−1.113 (*t*)	0.666
MI (F3, F4)	0.184 (0.163)	0.186 (0.304)	0.276 (U)	0.904
PLV (F3, F4)	0.241 (0.220)	0.247 (0.273)	0.457 (U)	0.864
PLI (F3, F4)	0.851 (0.266)	0.796 (0.228)	0.849 (U)	0.686
PCC (P3, P4)	0.415 ± 0.242	0.404 ± 0.267	0.158 (*t*)	0.948
MI (P3, P4)	0.269 (0.361)	0.228 (0.287)	0.913 (U)	0.722
PLV (P3, P4)	0.397 (0.355)	0.321 (0.290)	0.807 (U)	0.683
PLI (P3, P4)	0.803 (0.209)	0.842 (0.208)	−0.924 (U)	0.771
PCC (F3, P3)	0.435 ± 0.296	0.457 ± 0.242	−0.355 (*t*)	0.918
MI (F3, P3)	0.313 (0.409)	0.286 (0.348)	0.786 (U)	0.681
PLV (F3, P3)	0.442 ± 0.209	0.412 ± 0.203	0.583 (*t*)	0.790
PLI (F3, P3)	0.854 (0.416)	0.755 (0.257)	0.903 (U)	0.707
PCC (F4, P4)	0.412 ± 0.231	0.468 ± 0.226	−0.997 (*t*)	0.728
MI (F4, P4)	0.284 (0.315)	0.287 (0.346)	−0.181 (U)	0.948
PLV (F4, P4)	0.402 ± 0.185	0.422 ± 0.203	−0.404 (*t*)	0.894
PLI (F4, P4)	0.776 (0.181)	0.840 (0.178)	−0.319 (U)	0.929
PCC (F3, P4)	0.139 ± 0.334	0.272 ± 0.302	−1.718 (*t*)	0.360
MI (F3, P4)	0.174 (0.228)	0.187 (0.151)	0.149 (U)	0.936
PLV (F3, P4)	0.230 (0.252)	0.254 (0.222)	−0.117 (U)	0.943
PLI (F3, P4)	0.886 ± 0. 095	0.713 ± 0.135	5.537 (*t*)	**<0.001** ^*^
PCC (F4, P3)	0.206 ± 0.268	0.248 ± 0.318	−0.546 (*t*)	0.803
MI (F4, P3)	0.171 (0.166)	0.192 (0.228)	−0.595 (U)	0.797
PLV (F4, P3)	0.209 (0.238)	0.270 (0.220)	−0.701 (U)	0.739
PLI (F4, P3)	0.882 (0.348)	0.826 (0.204)	0.287 (U)	0.936

The symbol * indicates significance after correction. For EEG rhythms, powers or power ratios were calculated as frequency-domain features.

**Table 3:** Partial correlations between EEG feature variables and 60-day GOS, where RMS was used as a controlled variable.

Variable	Rho	Value of *p*
BSR	−0.331	**0.003** ^*^
Activity	0.077	0.496
δ	−0.118	0.299
θ	−0.111	0.328
α	−0.075	0.508
β	−0.077	0.498
β/α	−0.321	**0.004** ^*^
PLI (F3, P4)	0.224	**0.046** ^*^

^*^Significant results ≤ 0.05.

**Table 4:** Spearman’s correlations between EEG feature variables and 60-day GOS.

Variable	Rho	Value of *p*
BSR	−0.547	**<0.001** ^*^
RMS	0.578	**<0.001** ^*^
β/α	−0.422	**<0.001** ^*^
PLI (F3, P4)	0.264	**0.017** ^*^

^*^ Significant results ≤ 0.05.

**Figure 3:**
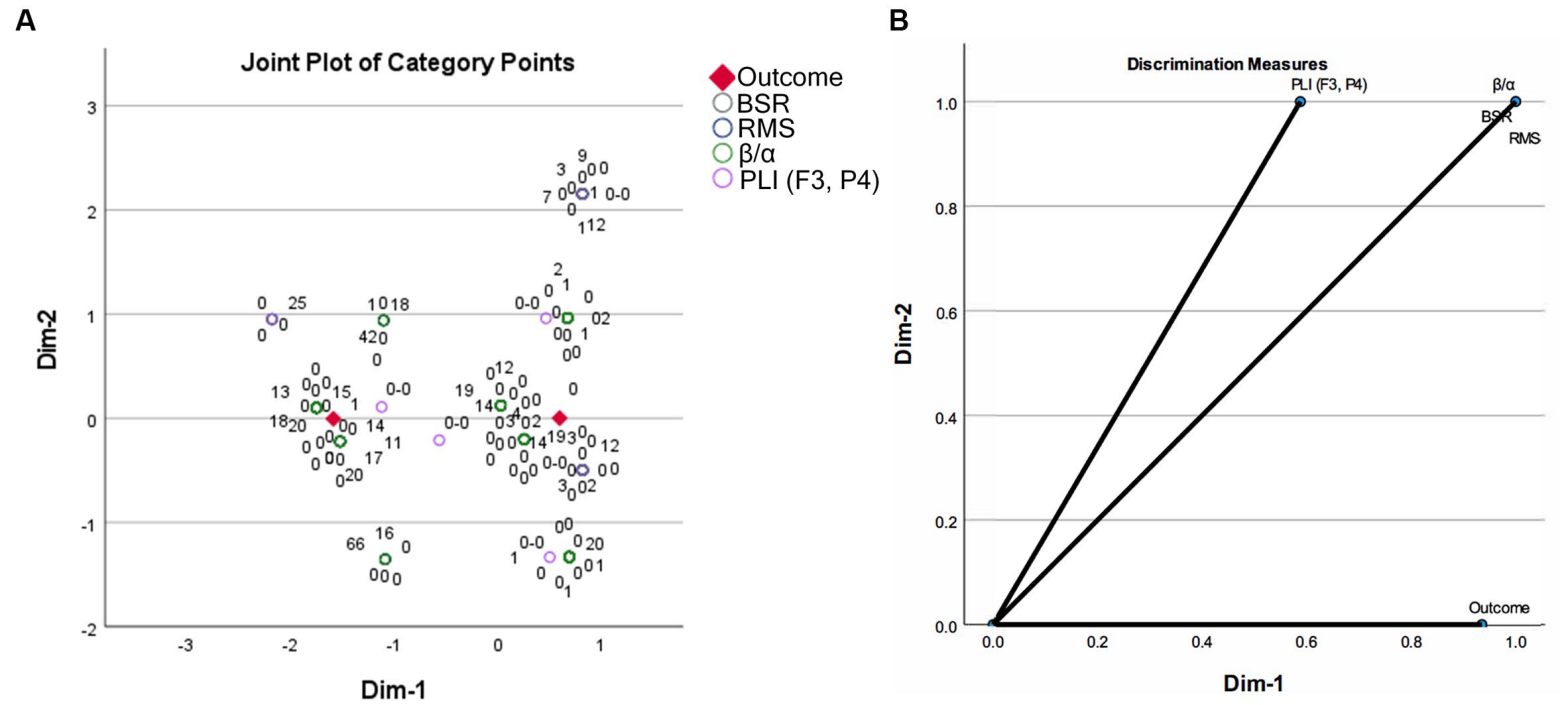
Multiple correspondence analysis results on the EEG features between two coma outcome groups: **(A)** joint plot of category points; **(B)** discrimination measures of all variables.

The task of establishing a prognosis model lies in discriminating between variables in two groups. Multiple correspondence analysis is a useful tool to map data from high-dimensional space to low-dimensional space, where visualization of discrimination among different groups can be achieved. The elements of row and column data in a contingency table are represented as category points in a 2-D space by MCA. In the output of MCA, the joint plot of category points shows separability of the corresponding variables belonging to different categories. If category points are close along some dimension or have close distances in the 2-D space, they are deemed to have close interactions. Discrimination measures give dimensional scores of category point separation, where a higher score implies that category points of a variable can be separated more easily along that dimension. The MCA results of the EEG features and the corresponding outcomes are presented in Figure 3. It can be found that, after being mapped to a 2-dimensional space, the four EEG features showed two discriminative clusters around the two coma outcomes along dimension 1. Note that the category points of BSR are perfectly overlapping with those of other features. Discrimination measures show that outcome can be separated along dimension 1, rather than dimension 2, and BSR, RMS, β/α, and PLI (F3, P4) all had high discrimination scores along dimension 1. This result implies that a combination of EEG features in various domains is promising to build a coma outcome predictor. The same discrimination score achieved by BSR, RMS, and β/α implies that these features may play similar roles in building a coma outcome predictor with combined features.

Logistic regression was performed to build coma outcome predictors using a single EEG feature or a combination of EEG features in various domains. Table 5 lists the results of single EEG feature-based unfavorable outcome predictors. It was found that BSR and β/α were risk factors, while RMS and PLI (F3, P4) were protective factors for predicting unfavorable outcome. All four features showed significance, i.e., AUC = 0.5 was rejected by Wilcoxon test of ranks. Multiple logistic regression for all four features was also performed, where likelihood ratio test was used to include or exclude variables. BSR and PLI (F3, P4) were reserved in the final prediction model, and the result is presented in Table 6. Partial correlation on EEG features with RMS was performed to adjust for interactions between frequency band powers and RMS, and hence powers of δ, θ, α, and β rhythms were excluded from building coma outcome predictors. Here we also performed multiple logistic regression with likelihood ratio test for all nine significant univariate features, and the result is identical to that in Table 6. Compared with the four significant features reserved after partial correlation, RMS and β/α were excluded from the final prediction model, which is also consistent with the MCA result where BSR, RMS, and β/α have identical discrimination scores along two dimensions.

**Table 5:** Logistic regression results of single features predicting unfavorable outcomes.

Variable	OR (95% CI)	Value of *p*
BSR	1041.015 (9.395–115,347.064)	**<0.001** ^*^
RMS	0.942 (0.908–0.978)	**<0.001** ^*^
β/α	5.021 (1.612–15.639)	**0.002** ^*^
PLI (F3, P4)	<0.001 (0.000–0.002)	**<0.001** ^*^

^*^ Significant results ≤ 0.05.

**Table 6:** Multiple logistic regression results of combining EEG features for predicting unfavorable outcomes.

Prediction model	Variable	OR (95% CI)	Value of *p*
BSR + PLI (F3, P4)	BSR	470.161 (2.872–76957.79)	**0.018** ^*^
	PLI (F3, P4)	<0.001 (0.000–0.008)	**<0.001** ^*^

^*^ Significant results ≤ 0.05.

**Figure 4:**
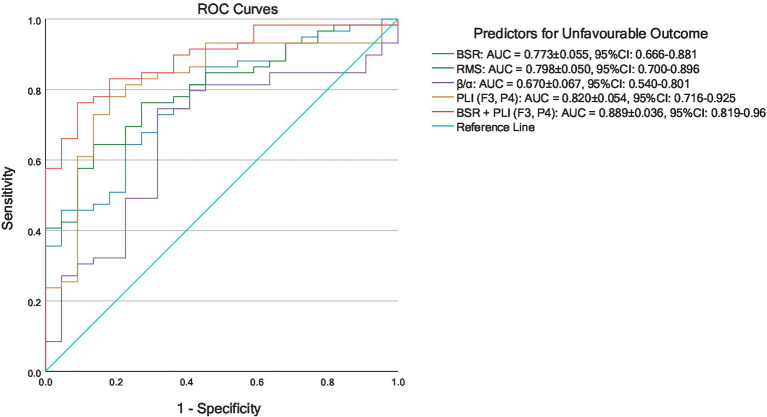
ROC curves of predictors for unfavorable coma outcomes established by 3-fold cross-validated logistic regression results.

The training and testing datasets were merged above in building coma outcome predictors, and hence the ROC curves reflecting prognostic performances were not considered in this combined cohort. To further illustrate the generalizability of the proposed method, *K*-fold cross-validation was performed. The involved 81 comatose patients were uniformly partitioned into *K* = 3 folds, and the distribution of outcome and etiology among 3 folds was as uniform as possible. In the i th round of cross-validation, the data in the i th fold formed the testing set, while the data in the rest folds were used for training the parameters of logistic regression. The prognostic characteristics of the coma outcome predictors built by EEG features in each round of 3-fold cross-validation are summarized in Table 7. It was found that in all rounds of cross-validation, all predictors achieved sensitivity ≥ 0.80 along with different levels of false positives. Among predictors using single EEG features, PLI (F3, P4) achieved the lowest false positives in all rounds of cross-validation. As expected, these single EEG feature-based predictors all suffered from medium to high false positives, indicating that cutoff probabilities can be set as larger than 0.5 for practical usage of these predictors, with degradation of sensitivity to some extent. It is noticed that the coma outcome predictor built by combining BSR and PLI (F3, P4) performed much better than those with single features in terms of low false positives. The cross-validated results on three testing datasets were merged, and then the comprehensive ROC curves of all predictors are plotted in Figure 4. Among all single variable-based predictors, PLI (F3, P4) achieved the largest AUC = 0.820 (95% CI: 0.716–0.925). Compared with these single variable-based predictors, the predictor with combined features, i.e., BSR + PLI (F3, P4), gave a larger AUC = 0.889 (95% CI: 0.819–0.960).

**Table 7:** Prognostic performance of predictors built by EEG features in 3-fold cross-validation.

Round of cross-validation	Predictors	Sensitivity (95% CI)	Specificity (95% CI)	PPV (95% CI)	NPV (95% CI)	False positives
1	BSR	0.85 (0.64–0.95)	0.14 (0.03–0.51)	0.74 (0.54–0.87)	0.25 (0.05–0.87)	0.86
	RMS	1.00 (0.84–1.00)	0.14 (0.03–0.51)	0.77 (0.58–0.89)	1.00 (0.21–1.00)	0.86
	β/α	0.80 (0.58–0.92)	0.00 (0.00–0.35)	0.70 (0.49–0.84)	0.00 (0.00–0.49)	1.00
	PLI (F3, P4)	1.00 (0.84–1.00)	0.57 (0.25–0.84)	0.87 (0.68–0.95)	1.00 (0.51–1.00)	0.43
	BSR + PLI (F3, P4)	0.95 (0.76–0.99)	0.71 (0.36–0.92)	0.90 (0.71–0.97)	0.83 (0.44–0.97)	0.29
2	BSR	0.95 (0.75–0.99)	0.25 (0.07–0.59)	0.75 (0.55–0.88)	0.67 (0.21–0.94)	0.75
	RMS	0.95 (0.75–0.99)	0.13 (0.02–0.47)	0.72 (0.52–0.86)	0.50 (0.09–0.91)	0.87
	β/α	0.95 (0.75–0.99)	0.13 (0.02–0.47)	0.72 (0.52–0.86)	0.50 (0.09–0.91)	0.87
	PLI (F3, P4)	0.79 (0.57–0.91)	0.38 (0.14–0.69)	0.75 (0.53–0.89)	0.43 (0.16–0.75)	0.62
	BSR + PLI (F3, P4)	0.89 (0.69–0.97)	0.63 (0.31–0.86)	0.85 (0.64–0.95)	0.71 (0.36–0.92)	0.37
3	BSR	0.85 (0.64–0.95)	0.57 (0.25–0.84)	0.85 (0.64–0.95)	0.57 (0.25–0.84)	0.43
	RMS	0.95 (0.76–0.99)	0.43 (0.16–0.75)	0.83 (0.63–0.93)	0.75 (0.30–0.95)	0.57
	β/α	0.85 (0.64–0.95)	0.57 (0.25–0.84)	0.85 (0.64–0.95)	0.57 (0.25–0.84)	0.43
	PLI (F3, P4)	0.80 (0.58–0.92)	0.71 (0.36–0.92)	0.89 (0.67–0.97)	0.56 (0.27–0.81)	0.29
	BSR + PLI (F3, P4)	0.80 (0.58–0.92)	0.86 (0.49–0.97)	0.94 (0.73–0.99)	0.60 (0.31–0.83)	0.14

### 3.2 Subgroup analysis

Subgroup analyses were performed on patients with strokes as well as patients with TBI. The stroke subgroup included 29 patients, of whom 6 had favorable outcomes and 23 had unfavorable outcomes. By comparison of EEG features between favorable and unfavorable outcome groups and then partial correlation analysis with RMS controlled, BSR, RMS, θ/total, θ/δ, and PLI (F3, P4) were preserved as significant parameters. The results are summarized in Table 8, and it was found that raised BSR, suppressed RMS, raised θ/total, raised θ/δ, or suppressed PLI (F3, P4) may be associated with unfavorable coma outcome for stroke patients. Due to the limited number of patients in the strokes subgroup, cross-validation was not carried out in this group, and the prognostic characteristics of the significant variables or combined model were not evaluated by logistic regression.

**Table 8:** Significant EEG features by comparison between favorable and unfavorable outcome groups in the stroke subgroup.

Variable	Favorable outcome (GOS ≥ 3)	Unfavorable outcome (GOS ≤ 2)	Statistical value	Value of *p* (after correction)
	*n* = 6	*n* = 23		
BSR	0.140 (0.061)	0.312 (0.172)	−3.284 (U)	<0.001^*^
RMS	38.765 ± 16.93	15.046 ± 8.46	4.902 (t)	<0.001^*^
θ/total	0.026 (0.013)	0.052 (0.071)	−3.069 (U)	0.048^*^
θ/δ	0.028 (0.016)	0.075 (0.118)	−2.907 (U)	0.016^*^
PLI (F3, P4)	0.877 ± 0.103	0.679 ± 0.130	3.455 (*t*)	0.019^*^

The symbol * indicates significance after correction. For EEG rhythms, powers or power ratios were calculated as frequency-domain features.

The TBI subgroup included 33 patients, of whom 11 had favorable outcomes and 22 had unfavorable outcomes. By comparison between favorable and unfavorable outcome groups, only PLI (F3, P4) was found significant (corrected value of *p* = 0.048) in the TBI subgroup, and the comparative details are summarized in Table 9. Due to the limited number of patients in the TBI subgroup, cross-validation logistic regression was also not performed, and the corresponding prognostic characteristics were not displayed here.

**Table 9:** The significant EEG feature derived by comparison between favorable and unfavorable outcome groups for analysis of the TBI subgroup.

Variable	Favorable outcome (GOS ≥ 3)	Unfavorable outcome (GOS ≤ 2)	Statistical value	Value of *p* (after correction)
	*n* = 11	*n* = 22		
PLI (F3, P4)	0.880 ± 0.083	0.711 ± 0.126	4.022 (*t*)	0.048^*^

The symbol ^*^ indicates significance after correction.

## 4 Discussion

A retrospective study on coma prognosis in ICU using EEG feature-based predictors was conducted, with a limited frontoparietal EEG montage. This study was motivated by a common real-world requirement of a limited-montage EEG coupled with an automated algorithm and an important role played by frontoparietal network in underlying recovery from coma (Wu et al., 2015). Continuous EEG is recommended for the monitoring of critically ill patients (André-Obadia et al., 2015), and ACNS EEG descriptors have been studied in prognosis of coma or DoC in the past decade. ACNS EEG descriptors not only include some continuous variables but also concern the occurrence of some pathological patterns. The persistence of electrical status epilepticus was usually used as one ACNS EEG descriptor (Hirsch et al., 2021) and has been associated with a greater mortality rate (Rossetti et al., 2005), while in this study low occurrences of epileptiform discharges coincided in both groups. Hence, in this study, detection of epileptiform pattern was not considered, which usually depends on visual inspection or artificial intelligence (AI)-assisted methods trained on a large dataset. Standard interpretation of some ACNS EEG patterns still remains difficult in visual inspection (Westhall et al., 2015). Instead, in this study, features were extracted by quantitative analysis, and the EEG features in temporal domain (BSR and RMS), frequency domain (β/α), and spatial domain (PLI [F3, P4]) all showed their prognostic values by analysis of all patients, with a limited frontoparietal EEG montage.

Timing of EEG recording after brain injury was considered important in the existing studies. In a prospective observational cohort study (Bauerschmidt et al., 2021), predictors established with quantitative EEG features at postanoxic days 4–6 achieved highest prediction accuracy. In our retrospective study, it was hard to control the EEG recording of all patients on the same days after brain injury. In spite of this, a comparison of timing of EEG recording between favorable and unfavorable outcome groups showed no significant difference, and the median EEG recording times are all within 1 week after brain injury.

In ACNS EEG descriptors, burst suppression and suppressed background EEG voltage (<10 μV) were usually considered as highly “malignant” patterns (Sandroni et al., 2014; Hofmeijer et al., 2016; Benarous et al., 2019; Backman et al., 2020; Guedes et al., 2020; Scarpino et al., 2020; Nolan et al., 2021; Hoedemaekers et al., 2023), which were associated with poor neurological outcome. The presence of burst suppression was associated with an unfavorable prognosis with 100% specificity (Benarous et al., 2019). A suppressed EEG was considered as a reliable predictor of poor outcome if it persisted beyond 24 h after the arrest (Hoedemaekers et al., 2023). At 72 h, an isoelectric, suppression, or burst suppression pattern on EEG predicted poor outcome with 100% specificity (Scarpino et al., 2020). According to our analytical results on all patients as well as the strokes subgroup, among all temporal features, raised BSR and diminished RMS (i.e., suppressed voltage or diminished EEG power) were predictors of unfavorable outcome. This result is highly consistent with the conclusions in the literature. It is also noticed that BSR + PLI (F3, P4) formed the ultimate predictor by multiple logistic regression on all patients, indicating that diminished EEG power may be a reliable but not the best predictor for unfavorable coma outcome with our limited frontoparietal EEG montage.

Among the rhythms (δ, θ, α, and β), dominant δ and θ oscillations and attenuated α oscillations were considered as indicators for unfavorable outcome (Benarous et al., 2019; Kustermann et al., 2019; Scarpino et al., 2020). For analysis on all patients, the powers of δ, θ, α, and β all showed significance in comparison between groups, while they were then discarded according to the results of partial correlation analysis. As attenuation and overall suppression are negative prognostic factors, decreased power across frequency bands in the unfavorable outcome group can be found. The raised power ratio β/α was found associated with unfavorable coma outcome. α oscillations were known to appear only during wakefulness, reflecting basal forebrain, thalamus, and cortical interactions (Babiloni et al., 2009). β oscillations, or higher frequency rhythms, were associated with enhanced alertness following the onset of activity in cholinergic aggregates of the brainstem and basal forebrain. In the favorable outcome group, raised α and β oscillations can all be found, and it seems that α oscillations were dominant compared to β oscillations. For the analysis of the strokes subgroup, raised θ/total and raised θ/δ were found associated with unfavorable coma outcome, which is consistent with the existing conclusions. It seems that dominant θ oscillations may play a more important role in coma prognosis of the strokes subgroup among all frequency-domain features. For the TBI subgroup, none of the frequency-domain features were found significant. This result may be attributed to that localization and extent of the brain damage of various TBI patients were not considered in this study. As aforementioned, because sedation may lead to decreased voltage, decreased slow wave, or raised fast rhythm, all EEG monitoring were initiated at or later than 24 h after short-acting low-dose sedating medications in our ICU. Even so, the influence of sedation after 24 h cannot be totally avoided, and the bias may still exist.

Functional connectivity metrics were used as spatial features. One study showed that postanoxic comatose patients with poor neurological outcome had less dynamics of brain functional connectivity (Keijzer et al., 2021). In Zubler et al. (2015), patients with recovery on day 10 showed higher coherence across various bands. The study in Carrasco-Gómez et al. (2021) showed that postanoxic comatose patients with raised PLV in θ and α rhythms at 12 and 24 h were associated with favorable outcome. In a recent study (Jiang et al., 2023), the whole-brain connectivity based on coherence, phase synchronization, PLI, and cross-correlation was found to be significantly enhanced for favorable outcome. Full montage used to be employed in past studies, while in our study, a limited frontoparietal montage including four EEG channels was used. It was shown that even with a small number of frontoparietal channels, there is still a significant functional connectivity metric, PLI (F3, P4), achieving best prognosis performance among features in all domains. As an indicator of favorable outcome, raised PLI (F3, P4) may reflect enhanced connection between bilateral hemispheres, as well as increasing connection between cortical/subcortical structures in frontal and parietal lobes. It is known that the metabolic activity and functional connectivity within the anterior forebrain mesocircuit and the frontoparietal network are associated with the restoration of cerebral activity (Edlow et al., 2021). Inspired by this discovery, though with a reduced EEG montage including only four channels, the calculated functional connectivity metrics can reflect the frontoparietal network activities. By analysis of all patients as well as the strokes and TBI subgroups, it was found that during recovery from coma, restoration of frontoparietal network may be accompanied by an enhanced PLI (F3, P4).

There are few studies involving a combination of automatically extracted EEG features in temporal, frequency, and spatial domains. The temporal- and frequency-domain features were combined to give predictive values of the revised cerebral recovery index (rCRI) in Nagaraj et al. (2018), where random forest classification was used. Features in frequency domain and spatial domain were considered in prognosis of coma in Jiang et al. (2023), but they were not combined to form a better predictor. A comprehensive study in Carrasco-Gómez et al. (2021) used COH, PLV, and MI as spatial features and combined non-coupling features in Nagaraj et al. (2018) for outcome prediction of comatose patients, achieving a sensitivity of 73% at 100% specificity. The above studies used full EEG montages, which were believed to be essential for functional connectivity analysis in the past. However, full-montage monitoring has also been recognized as labor-intensive and resource-demanding, making it inconvenient for practical applications. Enlightened by Backman et al. (2020), a reduced montage consisting of only four frontoparietal EEG channels was used for monitoring. Nevertheless, the results showed that features in all domains had their own prognostic value. Combination of features in various domains was found well suited to coma prognosis with reduced frontoparietal EEG montage, where the combination of BSR and PLI (F3, P4) brought great promotion of prognostic performance by analysis of all patients, compared with the predictors based on each feature alone.

The main limitation is that this study was a retrospective one. Whether and when a comatose patient should adopt bedside EEG monitoring was decided by the patient’s physician, and a bias risk of subject selection may occur. The included critically ill patients had mixed etiologies, and the sample size in each subgroup was also limited, hindering performance evaluation of the coma outcome predictors established by logistic regression in each subgroup. Additionally, for each patient, EEG recording was not performed at an identical time after admission. The future work would be a prospective study, in which EEG recording would be performed at specific time nodes, to investigate the best time for EEG monitoring for coma prognosis with our limited frontoparietal montage. The sample size in each subgroup still needs to be enlarged in future research to facilitate the development and performance evaluation of separate coma outcome predictors for each subgroup. Furthermore, as in cross-validations, most of the models yielded low to moderate specificities, i.e., clinically significant false positive rates for predicting unfavorable outcomes; further investigation is still needed to translate the findings into a clinically applicable model.

## 5 Conclusion

For purpose of practical applications of quantitative EEG monitoring in coma prognosis in ICU, a limited frontoparietal EEG montage was used in this study. By using only four frontoparietal channels of EEG recording, features in temporal, frequency, and spatial domains all found their own prognostic value for critically ill comatose patients. By cross-validation analysis on all patients, the combination of EEG features in multiple domains outperformed the prediction based on the feature in each of the domains alone. The verified prognostic value in this study may lead to an easy-to-implement quantitative assessment approach in ICU and hold valuable implications for future automatic coma prognosis applications if further investigation is performed to control the false positive rates at a low level.

## Data availability statement

The original contributions presented in the study are included in the article/supplementary material, further inquiries can be directed to the corresponding author.

## Ethics statement

The studies involving humans were approved by the First People’s Hospital of Kunshan Ethics Committee (Approval No. 00012098). The studies were conducted in accordance with the local legislation and institutional requirements. The participants provided their written informed consent to participate in this study.

## Author contributions

TT: Writing – original draft, Conceptualization, Investigation. SL: Conceptualization, Methodology. NH: Data curation, Methodology, Writing – review & editing. DX: Software, Methodology, Validation. CX: Formal Analysis, Resources. FL: Data curation, Investigation. QW: Formal Analysis, Visualization. YP: Writing – review & editing, Conceptualization, Funding acquisition, Project administration.
